# Relation between Length of Exposure to Epidural Analgesia during Labour and Birth Mode

**DOI:** 10.3390/ijerph16162928

**Published:** 2019-08-15

**Authors:** Laura Garcia-Lausin, Mercedes Perez-Botella, Xavier Duran, Maria Felisa Mamblona-Vicente, Maria Jesus Gutierrez-Martin, Eugenia Gómez de Enterria-Cuesta, Ramon Escuriet

**Affiliations:** 1 Department of Experimental and Health Science, Universitat Pompeu Fabra (UPF), 08003 Barcelona, Spain; 2 Parc de Salut Mar, 08003 Barcelona, Spain; 3 Research in Childbirth and Health Unit (ReaRH), University of Central Lancashire, 100, Picketlaw Road, G76 0BF Glasgow, UK; 4 Methodology and Biostatistics Support Unit, Institut Hospital del Mar d’Investigacions Mèdiques (IMIM), 08003 Barcelona, Spain; 5 Río Hortega University Hospital, 47012 Valladolid, Spain; 6 Centre for Research in Health and Economics, University Pompeu Fabra, 08005 Barcelona, Spain; 7 Catalan Health Service, Government of Catalonia, 08028 Barcelona, Spain; 8 Faculty of Health Sciences, University Ramon Llull-Blanquerna, 08025 Barcelona, Spain

**Keywords:** time exposure to epidural analgesia, alternative methods of pain relief, oxytocin, type of birth, experience of women, informed consent

## Abstract

Objective: To appraise the relationship between the length of exposure to epidural analgesia and the risk of non-spontaneous birth, and to identify additional risk factors. This study is framed within the MidconBirth project. Study design: A multicentre prospective study was conducted between July 2016 and November 2017 in three maternity hospitals in different Spanish regions. The independent variable of the study was the length of exposure to epidural analgesia, and the dependent variable was the type of birth in women with uncomplicated pregnancies. The data was analyzed separately by parity. A multivariate logistic regression was performed. The odds ratios (OR), using 95% confidence intervals (CI) were constructed. Main outcome measures: During the study period, 807 eligible women gave birth. Non-spontaneous births occurred in 29.37% of the sample, and 75.59% received oxytocin for augmentation of labour. The mean exposure length to epidural analgesia when non-spontaneous birth happened was 8.05 for primiparous and 6.32 for multiparous women (5.98 and 3.37 in spontaneous birth, respectively). A logistic regression showed the length of exposure to epidural during labour was the major predictor for non-spontaneous births in primiparous and multiparous women followed by use of oxytocin (multiparous group). Conclusions: The length of exposure to epidural analgesia during labour is associated with non-spontaneous births in our study. It highlights the need for practice change through the development of clinical guidelines, training programs for professionals and the continuity of midwifery care in order to support women to cope with labour pain using less invasive forms of analgesia. Women also need to be provided with evidence-based information.

## 1. Introduction

A woman who is giving birth´s response to labor pain depends on an array of factors such as, the type of onset of labour, a woman´s cultural background, antenatal class attendance, professional support during labor and medical interventions during labour, such as oxytocin use [1,2]. Several coping strategies for relieving pain in labour have been widely implemented and adequate evidence of the benefit in reducing pain exists for various non-pharmacological methods such as continuous labour support [2], relaxation techniques [3], water immersion [4], acupuncture [5], massage, transcutaneous electrical nerve stimulation (TENS) [6], intradermal water blocks [4], and maternal movement and positioning [7]. The evidence on pain relieving effects of pharmacological methods is also abundant, particularly for inhaled oxide nitrous [8], parenteral opioids [9]; and epidural analgesia (EA) [1]. The choice of pain relief depends on the woman’s preferences, and women can choose to use several of these methods during the course of labour. EA is an effective form of pain relief during labour (provides more effective pain relief than the other modalities) and is considered suitable for women once active labour has been established [10]. However, it is an invasive procedure with some side effects and, more rarely, serious complications.

EA has been shown [1] to be associated with several adverse outcomes with an absolute risk of 14.2% for assisted vaginal births, 12.2% for caesarean section, 1% for accidental dural puncture, 0.1% for severe allergic reactions, 21% for maternal long-term back-age, 2% for admission to neonatal intensive care units. EA interferes with the main hormones involved in the birth process, such as decreased oxytocin secretion, prostaglandin E2 or β-endorphins [11]. This hormone decrease leads to a longer labour, immobility, a greater need for stimulation with oxytocin for obstructed labour, a lengthened second stage of labour [12] and an increased incidence of fetal malposition during birth (occiput posterior and transverse positions) and the increased risk of an instrumental birth [1,13]. Women with epidural also have been described to experience more hypotension, motor blockade, fever, and urinary retention as demonstrated with overwhelming and good quality evidence [1,14]. A relationship has not been found between EA use and an increase in neonatal morbidity, usually expressed by means of low Apgar scores and pH values of umbilical artery [1].

This paper [1] provides an analysis of studies since 2005 which indicate that there is no longer an association between epidural analgesia and instrumental births, suggesting that modern approaches to epidural analgesia in labour where the use of lower concentrations of local anesthetic and more modern epidural techniques, such as patient-controlled epidural analgesia (PCEA) were more likely. This reduces the risk of suffering an instrumental birth associated with EA use during labour.

The quality evidence on the early initiation of EA in labour and the risk of instrumental birth seems to be contradictory as some studies support using early EA in labour (before the fetus is well down in the pelvis) is associated with an extension and difficulty in the flexion of the fetal head, which interferes with the rotation and descent and increases instrumental births [15,16]. However, a Cochrane systematic review (CSR) [13] suggests that women giving birth to their first baby found no differences in the risk of a caesarean section and an instrumental birth with early initiation versus late initiation of EA for pain relief during labour. Nonetheless, some limitations were found in the trials of this review, as they presented heterogeneity due to the following factors: Differing definitions of early and late epidural with regards to the degree of cervical dilatation; the technique of epidural analgesia; and the fact that various forms of alternative pain relief were given to women who were allocated in the late initiation of EA group to cover that period of delay, so that is it hard to assess the outcomes clearly. In addition, the participants on these trials were only primiparous women thereby limiting the generalizability of the findings.

Potential confounding factors for EA adverse effects exist and should be considered. Longer lasting delivery is itself a risk factor for instrumental vaginal delivery or even for cesarean sections. Further, as gestational anti-nociperception in longer periods of time could be exhausting and labour pain can become more severe, this results in more epidural analgesia requests. Furthermore, malpresentation is more painful for the woman in labour and therefore causes a slower labour that makes it more likely that the woman requests an epidural, and uses it for longer period and has an assisted delivery [17]. Additionally, cephalopelvic disproportion diagnosed during labour and suspected fetal distress during labour improves with tocolytics which makes labour longer. Therefore, a longer labour makes it more likely to have a longer exposure to EA and more chances to have an assisted delivery. These potential confounding factors bring to the forefront the question of whether EA causes more often instrumental births or if longer and more painful labour is a predictor of instrumental birth.

The admission to neonatal intensive care units could be associated with many different causes such as fetal distress during labour, fetal heart defects, prematurity and others. Further, there may be problems with breastfeeding as they are also associated with other factors as parenteral opioid administration during labour [18].

Additionally, other reasons that may explain these adverse effects include a series of physiological processes, associated health professionals’ responses and the interventions that can be considered part of the so called cascade of interventions [19,20]. The cascade of interventions is considered the unnecessary maternity care interventions that can lead to the need for more interventions with the potential to cause harm [21]. The literature has shown that women with uncomplicated pregnancies being cared for by professionals that let labour take its own pace (providing all is well with mother and baby), request less epidural analgesia and the rates of spontaneous vaginal births are higher [22]. There is existing evidence that the type of birth plays a crucial role in the women’s perceptions of their birth experience [3] and ultimately impacts on the health of the woman and the neonate [23]. 

Given that the aim of maternity care is to create a positive birth experience, minimizing adverse outcomes and helping to improve maternal and newborn health [24], then it is imperative that effective, sustainable and affordable intrapartum care practices are available to support women in labour to work with their labour and their pain as physiologically as possible. As a response, national and international agencies are calling for an improvement in intrapartum management of low risk women in spontaneous labour by using labour care practices that facilitate a physiologic labour process and minimize the intervention for appropriate women who are in spontaneous labour at term, and the importance of limiting these interventions to those who really need them only [11,25,26].

The National Health Service in Spain created the Strategy for Assistance in Normal Childbirth (SANC) [26] and some funding was allocated to facilitate the implementation of the policy. One of the main goals of SANC was to lower the rates of EA use during labour and recommended intrapartum care providers to adopt a restrictive policy for their use because of their potential harmful effects. To lower the rates of EA, SANC recommended a holistic approach to labour pain management discussions during the antenatal period, exploring alternative pain relief methods and emphasizing the women’s own ability to secrete endorphins (which have an analgesic effect) when conditions of intimacy and a feeling of safety are present during labour [27]. In addition, midwives and gynecologists are called to discuss fully with the mother in the antenatal period the benefits and potential risks of EA for the mother, fetus and baby and are asked to avoid using the procedure routinely and whenever possible, EA without a motor block.

In 2012, the National Health System in Spain evaluated the implementation of the normal birth policy SANC and estimated that EA was still used in 72.2% of the cases, higher than in other western countries; 30% in the United Kingdom, 60% in the US, 30–69% in Canada [1]. Oxytocin use was shown to be also very high (53.3%) despite national and international recommendations endorsing a much lower rate of 5 to10% of recommended oxytocin use [11,25].

In 2017, the authors decided to conduct the MidconBirth (MCB) study [28] which aimed to analyze the midwives’ contribution to normal childbirth care by assessing prospectively labour progress, birth practices carried out by the specific health professional (Midwife or Obstetrician) and assessing birth results.

As part of this study, a new line of research was introduced where the main aim was to investigate whether there was association between the length of the mothers’ EA exposure during labour and the birth mode in the sample of the study. The hypothesis formulated was that a long exposure to EA is one of the factors associated with non-spontaneous vaginal birth.

## 2. Material and Methods

This was a multicentre prospective study. The recruitment occurred between July 2016 and November 2017 in three hospitals in different Spanish regions (Virgen de los Lirios Hospital in Alcoi, Alicante, Rio Ortega University Hospital in Valladolid and Hospital del Mar in Barcelona). The inclusion and exclusion criteria are presented (Table 1).

### 2.1. Sample

The sample was limited to live singleton, spontaneous vaginal births, instrumental vaginal births and caesarean section deliveries, in primiparous and multiparous women between 37 and 42 weeks of gestation. The women were between 18 and 40 years of age with uncomplicated pregnancies. All the participants in the study were exposed to EA during labour. This study population included 807 from the 1260 women who gave birth in this period in the three participating hospitals. Further, 264 women were excluded from the study as they did not use EA during labour and 46 childbearing women had type I or urgent caesarean sections due to fetal distress [29] or elective caesarean section and were also excluded, and 143 women had missing observations in the register and had to be excluded from the study. For this study, type I or urgent caesarean section with less than two hours of epidural exposure were excluded. This study decided to use two hours as the cut-off point as the mean exposure to epidural analgesia was 5.51 (DS), and so it was considered that less than two hours was probably too short to give birth spontaneously and to have an impact. Women having a caesarean section (CS) due to obstructed labour or due to fetal distress with more than two hours of EA exposure were included in the study.

### 2.2. Variables of the Study

The dependent variable in this study was the mode of birth (spontaneous vaginal birth/non-spontaneous vaginal birth—including instrumental vaginal birth and caesarean section). The independent variable was the length of exposure to EA during labour. The participant hospitals employed the same EA regime for labour: 0.0625% bupivacaine plus 2 mg of fentanyl with patient controlled epidural analgesia (PCEA). The maternal, demographic, clinical factors and birth characteristics in the study were compared: The mother´s age, the weight type of the onset of labour oxytocin use, the gestational age, the birth mode and the newborn.

### 2.3. Data Collection

The data was recorded by the midwives in each participant hospital using a pre-designed standardized data collection sheet and the data was introduced into the MCB electronic database. The information was obtained from the three maternity units in different regions of Spain. Each midwife registered data from all childbearing women with uncomplicated pregnancies who met the study criteria until a representative sample was achieved for each hospital. The detailed data was forwarded on a daily basis from the medical records system to the electronic MCB database which contains information from July 2016 onwards. 

### 2.4. Analysis and Statistics

To overcome the confounding effect of parity, primiparous and multiparous at term were segregated, as the duration of labour tends to be different in first labour than in subsequent labours [10]. This segregation helped to investigate the factors associated with non-spontaneous births in each cohort of women.

The descriptive analysis of the sample used percentages and frequency distributions for categorical variables and the means for quantitative continuous variables.

The univariate analysis was used to check the association of obstetric characteristics with non-spontaneous births. The qualitative variables were compared by a Chi-square or Fisher exact test, as appropriate, and the quantitative variables by the student´s *t*-test. Considering the dichotomous outcome (spontaneous vaginal birth/non-spontaneous birth), logistic regressions were performed in order to determine which of the covariates were associated with non-spontaneous births. These associations were expressed by odds ratios (OR). The univariate and multivariate analysis were used to examine the associations between the outcome and covariates. Further, *p* values less than 0.05 were considered as statistically significant. The statistical analysis was performed using STATA Version 15 (STATA Corp., College Station, TX, USA).

### 2.5. Ethical Considerations

This study was approved by the ethics committee of the coordinating center (Clinical Research Ethics Committee of Parc de Salut Mar 2016/6785/I) and later by the ethics committee of each participating center. Informed consent from women was not required because no intervention other than usual care was performed, and only anonymized data was collected. 

## 3. Findings

### 3.1. Socio-Economic Maternal Background Data

The mean age of the women in the study was 31.93 years (SD = 5.13) with a mean gestational age of 40 weeks (33.09%), (Table 2).

A total of 807 women (75%) used epidural analgesia (EA) as a form of pain relief during labour in the three maternity units during the study period. The spontaneous onset of labour occurred in 68.28% of the sample, and 31.72% had an induction of labour. Further, 75.59% of the women in the study received oxytocin for augmentation of labour and 70.63% of the women in the sample had spontaneous vaginal births, and 29.37% had a non-spontaneous vaginal birth. From this, 17.47% had instrumental vaginal births (6.57% vacuum delivery, 10.9% forceps delivery) and 11.9% had a caesarean section. In the sample, there was a higher rate of primiparous women (56.26% versus 43.74% multiparous women) (Table 2). The mean length of the exposure to EA during labour was 5.51 h (SD = 3.50). 

### 3.2. Outcomes

After segregation of the sample by parity, Table 3 shows that the mean length of the exposure in primiparous women having a spontaneous vaginal birth was 5.98 h (SD = 2.91) and in multiparous women, this was 3.37 h (SD = 2.19). With regards to non-spontaneous vaginal births, the mean exposure length in primiparous women was 8.05 h (SD = 3.87) and in multiparous, this was 6.32 h (SD = 3.94). The exposure length to EA was higher in the non-spontaneous birth group and it was consistent between the two groups (primiparous and multiparous). The length of exposure to EA did not have an impact on the incidence of caesarean sections. The exposure length to EA was higher in the non-spontaneous vaginal birth group and had statistical significance in both primiparous and multiparous women (*p* < 0.001 in both groups). 

The bivariate analysis showed that oxytocin use for labour augmentation in primiparous women who had a spontaneous vaginal birth was 81.23% of the cases, and 87.57% in primiparous women with a non-spontaneous birth (*p*-value: 0.006). For multiparous women with spontaneous vaginal births, oxytocin for augmentation was used in 60.75% of the cases, and in 86.67% of those with a non-spontaneous birth (*p*-value-0.001).

Table 4 presents the multivariate logistic regression which shows the effect of the different factors (exposure time to EA, mother´s age, oxytocin use, gestational age, newborn weight, type of onset of labour) in each type of birth (spontaneous vaginal birth/ non-spontaneous birth) after adjusting for parity. The results showed that the length of the exposure to EA in primiparous as well as in multiparous women was associated with non-spontaneous births, being statistically significant (OR 1.20; CI 95%: 1.12–1.28, *p*-0.00; OR 1.40; CI 95%: 1.25–1.56, *p*-0.00 respectively).

It has been shown than multiparous women in this study were less exposed to the risk of experiencing non-spontaneous vaginal births compared with primiparous women [30], but when multiparous women in the study were administered oxytocin, they were significantly at greater risk of suffering non-spontaneous vaginal birth. (OR 3.05; CI 95%: 1.28–7.30, *p*-0.01) vs (OR 1.23; CI 95%: 0.68–2.24, *p*-0.49).

Logistic regression showed that the variable length of exposure to EA was the strongest factor associated with non-spontaneous birth compared with the other co-variates, even when compared with oxytocin use in multiparous women.

## 4. Discussion

This study focused on analyzing the length of exposure to EA alongside different confounding factors believed to contribute to non-spontaneous births. When confounders such as the mother´s age, oxytocin use, gestational age, newborn weight and type of onset of labour were included in this analysis, the length of exposure to EA in the study sample remained associated with non- spontaneous births. The conceptual scope of this study is explanatory, not predictive, therefore, our results do not prove a causal association, and there may be other potential confounding factors that could influence the mode of birth. However, no such extended data was collected because the aim of this study was to study the impact of the length of exposure to EA on the mode of birth. The main finding observed in this study was that women had more non-spontaneous births when exposure to EA was higher than 8.05 h in primiparous women and higher than 6.32 in multiparous women. This study provides the incidence rates of non-spontaneous vaginal births in this setting and identifies various risk factors and practice areas which contribute to non-spontaneous births. These data show that women in the sample who were exposed for longer periods to EA suffered more non-spontaneous births. An overuse of EA is seen in this study as 75% of the populations used EA during labour which is much higher than in other countries (e.g., 30% in the UK, 60% in the US, 30–69% in Canada [1]. It is plausible to assume that this high rate of EA use is at least partly attributable to the medical obstetric practice that is predominant in Spain and which tends to be highly interventionist [31]. Even though EA use is not a direct cause of non-spontaneous birth (it is more likely to be multifactorial), it is part of the cascade of interventions that may lead to this and so it should be avoided whenever possible. This agrees with the recommendations by the Lancet’s Maternal Health series which highlights the detrimental effects of overusing technology in developed countries [32]. The logistic regression found that the length of exposure to EA in this sample was clearly associated with higher rates of non-spontaneous births. Nevertheless, there are many factors acknowledged that make women have longer labours, therefore longer exposure to EA and more chances to have a non-spontaneous birth. The delayed first or second stage of labour, fetal malpresentations, cephalopelvic disproportion, suspected fetal distress and tocolytics´ use that make labour longer, and therefore longer exposure to EA and more risk of non-spontaneous births. On the other hand, it is unquestioned and has to be considered that interventional delivery may be required to improve birth outcomes when the situations where the mother´s life and/or the fetus´s life are threatened. For example, uterine rupture, fetal distress, chorioamnionitis, obstructed labour, placental abruption, placental detachment, cord prolapse and other emergency situations, where having a non-spontaneous vaginal birth is beneficial and intervening improves perinatal morbidity.

However, our study identified an association between the use of oxytocin and non-spontaneous birth which corroborates existing abundant literature in this regard [33]. In our study, 75% of women were administered with oxytocin. This high rate of oxytocin use may be partly explicated by an over-use of the procedure in the study´s clinical settings, employed even in the absence of medical indications. Oxytocin use is considered beneficial in the case of the delayed first stage of labour, although oxytocin may be used in situations when it is not beneficial. For, intrapartum care for healthy women and babies, the NICE guidelines recommend not to routinely offer interventions (such as active management of labour, amniotomy and oxytocin) during the first stage of labour [10]. Therefore, the guidelines for labour dystocia and augmentation, based on the latest evidence should be followed, allowing labour to progress uninterrupted, and respecting its normal progress without rushing it, especially in view of Gaudernack et al.’s study which shows the association between the use of oxytocin and instrumental births [30]. The NICE guidelines recommend to actively intervene in very specific cases where labour delay is confirmed (i.e., if delay in first stage of labour with ruptured membranes if cervical dilatation less than 2 cm in 4 h, NICE guidelines recommends starting oxytocin) . The high rate of oxytocin found in our study settings (75%) suggested that oxytocin was used generally in situations where it was not necessarily beneficial as per the guidelines’ recommendations [26]. The use of oxytocin for women without an identified indication has been questioned and Wei et al. (2013) showed that its use is not beneficial when compared with expectant management, as it carries risks that include uterine hyperstimulation and fetal distress [34]. For the purpose of our study, the data was not collected to determine whether oxytocin was used following NICE guidelines’ recommendations or not, but the high rate of oxytocin found in our study settings suggests that oxytocin was used in a liberal manner. The three participant hospitals in this study had similar oxytocin rates, and this was associated with non-spontaneous births in multiparous women. Consequently, and supported by the literature, the use of oxytocin should be reframed under a policy supporting a more restrictive approach. Understanding and believing that progress of labour can be negatively affected by long exposure to EA and oxytocin use should allow professionals to adopt a more measured approach to assessing the progress of labour [35].

When a woman chooses to use EA during labour, it is not possible to predict how many hours she will need it, as labour progress can vary and is affected by many factors. However, there is evidence on how other methods of pain relief (both pharmacological but interestingly non-pharmacological also) help women cope with labor pain in the first and second stages of labour. It seems reasonable therefore to suggest that providing women with a wide variety of pain coping and comfort measures, can help reduce the rates of EA, especially since, as shown by this and other studies, EA is associated with the increased use of oxytocin, both central elements in the so called cascade of obstetric interventions [19]. However, the alternatives to EA should be widely available known in advanced to the woman, and health professionals should be fully conversant with those methods. Overall, women should feel free to choose whatever pain management they think would help them most during labour, and those who choose non-drug pain management should be able, if needed, to move onto a drug intervention. Nonetheless, professionals should try to support the woman for as long as possible using less invasive methods of pain relief providing the woman has not expressed a strong preference for EA. This is because of the impossibility to predict the duration of exposure to EA and because of EA´s association with more negative outcomes [1]. For those instances when EA is needed, the promotion of modern combined spinal-epidural analgesia regimes (mobile epidural) have been shown to improve birth outcomes and reduce the rates of instrumental vaginal births [12]. 

Within this backdrop in Spain, it is welcome that the Lancet Midwifery Series [35] described evidence-based effective midwifery interventions helpful in labour to promote healthy physiologic labour as relaxation techniques and inhaled analgesia for pain relief in labour, immersion in water in the first and second stages, upright positions, massage, reflexology, acupuncture or acupressure. In the same line, a global initiative (International Childbirth Initiative-ICI, 2018) [36] was developed. This initiative has a mother-baby-family maternity care model that shifts the traditional medical model of care to a value-based model grounded in partnership between the provider and users, where optimal health outcomes are the main goal. Some of the recommendations from ICI initiative which could address the concerns raised by the authors study is offering drug-free, comfort and pain relief measures as a safe first option, explaining their benefits for facilitating normal physiologic birth. If pharmacological pain relief options are requested, their benefits and risks should be explained. Another related recommendation from ICI stands for providing evidence-based practice as it has been proven to be beneficial in supporting the normal physiology of birth. Therefore, there appears to be a lack of adherence to national and international recommendations to adopt strategies that facilitate labour physiology [10,25,26,36] in favor of an interventionist approach of care provision during labour, symptomatic of a medicalized model of maternity care which has the potential to result in care that is more expensive. Therefore, addressing the rates of the length of exposure to EA and oxytocin use during labour is an important consideration when striving to improve women´s health. The current predominant medical model of care in Spain could benefit from adopting these strategies to help improve childbirth perinatal results. 

It would be beneficial in future studies to investigate and discuss other potential confounding factors, such as the indication for oxytocin if used, the differences in outcomes according to oxytocin indications, the complications during labour as malposition, fetal complications or concerns. Further, to take into account and discuss other factors, as for example, low Apgar scores, babies requiring admission to NICU (neonatal intensive care unit), neonatal sepsis or hypoxia and perineal tears. Thus, being able to compare the differences in these factors between those with longer EA exposure, those without long exposure to EA and those without EA exposure.

Subsequently, it would be beneficial that further studies examined women’s satisfaction with the use of EA in a context where other methods of pain relief are available and widely used. Also, randomized controlled trials should be conducted in order to continue advancing knowledge on the impact that EA has on childbirth.

### 4.1. Strengths of the Study

This observational study plays a significant role in health research, particularly as evidence from randomized controlled trials is not available in this area. This is the first study which analyzes the length of exposure to EA associated with birth results in women with uncomplicated pregnancies. 

This study has identified factors relating to obstetric and midwifery practices which have the potential to minimize non-spontaneous vaginal births rates and the length of exposure to EA. With this study, it has been possible to build a regression to see the association of each variable with the outcome (type of birth) after adjusting for the effects of other variables (mother´s age, use of oxytocin, type of onset of labour), and length of exposure to EA has shown to be the strongest predictor for a non- spontaneous vaginal birth. The clinical information derived from our study is a major strength as it provides an insight into current obstetric practice in a large and diverse population.

### 4.2. Limitations

The management of labour at the three hospitals that participated in the study may differ from other regions, thereby limiting the generalizability of our findings. For this prospective study, some of the data collected for the MidconBirth study was used and some specific data to be collected was added. However, not all the relevant information was available for the analysis as for example, the information about cervical dilation when EA was placed or for other confounding factors. This study included women with the induction of labour, and this group could have different outcomes compared with the spontaneous onset of labour group.

## 5. Implication for Practice and Conclusions

The contributory factors that lead to a birth resulting in a non-spontaneous birth have been shown to be multifactorial [37]. Our results suggest that the length of exposure to EA influences the birth mode. In addition, it was noted that in our study settings, there was a high rate of oxytocin use (75%).

This study has identified three main ways to tackle the above. There are Educational packages for professionals, a real alternative to EA for pain relief in labour and the continuity of midwifery care in labour [19,25]. The education packages for all providers of maternity care should focus on the importance of meeting the holistic needs of mothers and families [36]. Care professionals should receive training underpinned by the midwifery philosophy of care [35] in all comfort measures and pain relief options with the ultimate aim to develop philosophies of care that respect women’s preferences and informed choices to maximize their confidence and wellbeing. The Education to women (and their companions) about physiological labour and on alternative pain relief methods (including their benefits and risks) should also be a routine part of antenatal care.

Lastly, the results of the present study should prompt policy makers, organizations, managers and professionals to think about ways in which they can enhance care provision by facilitating continuity of midwifery care. Midwifery care in the three participant hospitals was not one to one care during labour. The midwives in the three participant hospitals look after two or three women in labour at a time, not being possible to give continuity of midwifery care. The authentic and continued support from the midwife reduces the need for analgesia in labour and improves birth outcomes [38,39,40]. Since midwives are essential in contributing to high-quality maternal and newborn services [40], increasing a midwifery presence during labour appears to be a strategy that would enhance maternal and newborn health as well as probably having a positive important economic impact, potentially reducing health spending [41]. 

## Figures and Tables

**Table 1:** The inclusion/exclusion study criteria.

Inclusion Criteria	Exclusion Criteria
Having had an EA during labour	type I or urgent caesarean section (with <2 h EA exposure during labour)
	Elective caesarean section
Spontaneous vaginal births	Not having had an EA during labour
Induced vaginal births	Birth of a live singleton infant in breech presentation
Instrumental vaginal births	Complicated pregnancies
Type II lower segment Caesarean sections	Gestational age under 37 and above 41 weeks gestation
Births of a live singleton infant in cephalic presentation with uncomplicated pregnancies	Women age under 18 or above 40 years of age
Women in labour using EA	
Gestational age between 37 and 41 weeks’ gestation	
Women between 18 and 40 years of age	

EA: Epidural Analgesia.

**Table 2:** The maternal, fetal and birth characteristics among women with epidural analgesia.

Variables	
Time exposure to epidural analgesia: mean (SD)	5.51 (3.50)
Maternal age: mean (SD)	31.93 (5.13)
	n (%)
Oxytocin use	
No	197 (24,41)
Yes	610 (75,59)
Type of birth	
Spontaneous vaginal birth	570 (70.63)
Vaginal birth, Vacuum	53 (6.57)
Vaginal birth, Spatulas	28 (3.47)
Vaginal birth, Forceps	60 (7.43)
Cesarean section	96 (11.9)
Parity	
Primiparous	454 (56.26)
Multiparous	353 (43.74)
Gestational age (weeks gestation)	
37	45 (5.58)
38	128 (15.86)
39	215 (26.64)
40	267 (33.09)
41	152 (18.84)
Newborn birth weight (grams)	
<2500	14 (1.73)
2501–3000	180 (22.30)
3001–3500	375 (46.47)
3501–4000	197 (24,41)
>4000	41 (5.08)
Type of labour onset	
Spontaneous	551 (68.28)
Induction	256 (31.72)

SD: standard deviation; n: sample.

**Table 3:** The risk of non-spontaneous birth, maternal, fetal and birth characteristics by parity.

	Primiparous			Multiparous		
	SVB	Non-SB	*p*-Value	SVB	Non-SB	*p*-Value
Time exposure to epidural analgesia: mean (SD)	5.98 (2.91)	8.05 (3.87)	*p* < 0.001	3.37 (2.19)	6.32 (3.94)	*p* < 0.001
Mother´s age: Mean (SD)	30.58 (0.32)	31.98 (0.38)	*p*-0.006	32.93 (0.27)	33.18 (0.62)	*p*-0.703
	n (%)	n (%)		n (%)	n (%)	
Oxytocin Use						
No	52 (18.77)	22 (12.43)		115 (39.2)	8 (13.33)	
Yes	225 (81.23)	155 (87.57)	*p*-0.074	178 (60.75)	52 (86.67)	*p* < 0.001
Gestational age (weeks gestation)						
37	19 (6.86)	10 (5.65)		15 (5.12)	1 (1.67)	
38	53 (19.13)	18 (10.17)		49 (16.72)	8 (13.33)	
39	66 (23.83)	44 (24.86)		85 (29.01)	20 (33.33)	
40	88 (31.77)	64 (36.16)		99 (33.79)	16 (26.67)	
41	51 (18.41)	41 (23.16)	*p*-0.104	45 (15.36)	15 (25.00)	*p*-0.242
Newborn birth weight (in grams)						
<2500	6 (2.17)	3 (1.69)		4 (1.37)	1 (1.67)	
2501–3000	70 (25.27)	39 (22.03)		57 (19.45)	14 (23.33)	
3001–3500	136 (49.10)	81 (45.76)		132 (45.05)	26 (43.33)	
3501–4000	57 (20.58)	43 (24.29)		81 (27.65)	16 (26.67)	
>4000	8 (2.89)	11 (6.21)	*p*-0.357	19 (6.48)	3 (5.00)	*p*-0.960
Type of onset of labour						
Spontaneous	191 (68.95)	103 (58.19)		221 (75.43)	36 (60.00)	
Induction	86 (31.05)	74 (41.81)	*p*-0.019	72 (24.57)	24 (40.00)	*p*-0.014

SVB: spontaneous vaginal birth; Non-SB: spontaneous birth.

**Table 4:** The adjusted OR non-spontaneous birth by parity.

	Primiparous Women		Multiparous Women	
	OR (95% CI)	*p*-Value	OR (95% CI)	*p*-Value
Exposure time to epidural analgesia	1.20 (1.12–1.28)	<0.001	1.40 (1.25–1.56)	<0.001
Maternal age	1.04 (1.00–1.09)	0.034	1.02 (0.96–1.10)	0.479
Oxytocin use	1.23 (0.68–2.24)	0.498	3.05 (1.28–7.30)	0.0 12
Type of onset of labour				
Induction	1.43 (0.92–2.22)	0.112	1.47 (0.74–2.89)	0.2 71

OR: Odds Ratio; CI 95%: Confidence Interval 95%.
